# International meeting on research advances and rabies control in the Americas.

**DOI:** 10.3201/eid0203.960318

**Published:** 1996

**Authors:** C E Rupprecht


					Vol. 3, No. 2--July-September 1996 Emerging Infectious Diseases
243
News and Notes
International Meeting on Research
Advances and Rabies Control in the
Americas
The "VIIth, Annual International Meeting on Re-
search Advances and Rabies Control in the
Americas" will be convened at the Centers for Dis-
ease Control and Prevention in Atlanta during 9-13
December 1996. The Conference will focus on both
basic scientific contributions and applied research
in the understanding and control of this fatal viral
disease. Conference sessions are designed fro a di-
verse public health and biomedical audience,
particularly professionals in Canada, the United
States, and Latin America; the sessions will explore
research advances, diagnostic issues, viral path-
obiology, oral immunization, descriptive and
molecular epizootiology, host ecology, and human
preexposure and postexposure vaccination. Work-
shops detailing techniques used in the diagnosis and
surveillance of rabies by conventional and molecu-
lar methods will also be held.
For further information, contact
Dr. Charles E. Rupprecht
Chair, Organizing Committee
Centers for Disease Control and Prevention
1600 Clifton Road, MS G33
Atlanta, GA 30333, USA
fax: 404-639-1058
e-mail: cyr5@ciddvd1.em.ced.gov.
Symposium Notice
The Infectious Disease Association of California
(IDAC) will hold its fall symposium on Saturday,
October 19, 1996, at the Hyatt Regency in Irvine,
California.
Registration, including lunch, is free to IDAC
members; $50 for non-members.
For information, call
(310) 398-5931 or e-mail: IDAC@aol.com.

				

